# New, Cheap and Self-generating Disinfectant

**Published:** 1878-09

**Authors:** 


### A New, Cheap and Self-generating Disinfectant.

Under this title, Dr. John Day, of Geelong, Australia, recommends for use in civil and military hospitals, and also for the purpose of destroying the poison-germs of infectious diseases, a disinfectant composed of one part of rectified oil of turpentine and seven parts of benzine, with the addition of five drops of oil of verbena to each ounce. Its purifying and disinfecting properties are due to the power presumed to be possessed by each of its ingredients, of absorbing atmospheric oxygen, and converting it into either peroxide of hydrogen, or ozone. Articles of clothing, furniture, wall-paper, carpeting, books, newspapers, letters, etc., may be perfectly saturated with it without receiving the slightest injury, and when it has been once freely applied to any rough or porous surface, its action will be persistent for an almost indefinite period. This may, at any time, be readily shown by pouring a few drops of a solution of iodide of potassium over the material which has been disinfected, when the peroxide of hydrogen, which is being continually generated within it, will quickly liberate the iodine from its combination with the potassium, and give rise to dark-brown stains. It may be applied with a brush or a sponge, or, if more convenient, as is the case with certain articles, such as books, newspapers and letters, it may be simply poured over them until they are well soaked; they may then be allowed to dry, either in a warm room or in the open air.—*New Remedies.*

